# Have We Met Before? Findings on HIV Retesting in Routine Healthcare Settings, Using Eswatini HIV‐1 Recent Infection Surveillance Data

**DOI:** 10.1002/jia2.70187

**Published:** 2026-09-16

**Authors:** Claire C. Steiner, Eugenie A. Poirot, Samkelo Simelane, Dumile Sibandze, Lenhle Dube, Vusie Lokotfwako, Jessica Justman, Kathryn Lupoli, Trudy Dobbs, Munyaradzi Pasipamire, Cebisile Ngcamphalala, Ruben Sahabo, Harriet Nuwagaba‐Biribonwoha, Suzue Saito

**Affiliations:** ^1^ Mailman School of Public Health ICAP at Columbia University Lusaka Zambia; ^2^ Mailman School of Public Health ICAP at Columbia University New York United States; ^3^ Mailman School of Public Health ICAP at Columbia University Mbabane Kingdom of Eswatini; ^4^ Ministry of Health Government of the Kingdom of Eswatini Mbabane Kingdom of Eswatini; ^5^ U.S. Centers for Disease Control and Prevention Atlanta Georgia USA; ^6^ U.S. Centers for Disease Control and Prevention Mbabane Kingdom of Eswatini; ^7^ Department of Epidemiology Mailman School of Public Health Columbia University New York New York USA

**Keywords:** HIV, HIV‐testing, non‐disclosure of ART, repeat HIV‐testing, retesting, surveillance

## Abstract

**Introduction:**

Eswatini is rapidly approaching the conditions needed to achieve and sustain HIV epidemic control. Repeating diagnostic testing of people living with HIV (PLHIV) already on antiretroviral therapy (ART) is not typically indicated, can lead to misidentification of new HIV diagnoses and distorts estimates of progress towards epidemic control. This study aimed to quantify retesting among PLHIV on ART and identify associated characteristics, using biomarker‐based Recent Infection Surveillance data from routine HIV testing in Eswatini.

**Methods:**

The Eswatini HIV‐1 Recent Infection Surveillance (EHRIS) programme, launched in July 2019, offered a rapid test for recent infection (RTRI) to PLHIV 15 years and older who tested HIV‐positive during routine HIV testing. We analysed survey data collected from 208 HIV testing locations (facility‐ and community‐based) between July 2019 and February 2024. The analysis included data of PLHIV who did not disclose a prior HIV diagnosis during pre‐test counselling and subsequently underwent an RTRI. We defined retesting among PLHIV with RTRI recent and VL <1000 copies/mL. Data were analysed to evaluate characteristics of retesting using Rao−Scott chi‐square, bivariable and multivariable logistic regression.

**Results:**

Of 1925 adults who provided blood specimens for RTRI and VL testing, 781 (40.6%: 95% CI, 37.1–44.1) had suppressed VLs and were classified as retesting and likely on ART. The adjusted odds ratio (aOR) of retesting was higher if testing occurred at community locations (aOR = 1.9, *p* = 0.0007), during periods of more restrictive stay‐at‐home policy (aOR = 1.7, *p* = 0.005, aOR 2.6, *p*<0.0001, aOR = 2.1, *p*<0.0001), among females (aOR = 1.6, *p* = 0.002), pregnant females (aOR = 1.8, *p* = 0.001), co‐habiting (aOR = 1.8, *p* = 0.02) or self‐reported prior HIV testing (aOR = 1.3, *p* = 0.03).·

**Conclusions:**

Retests for HIV account for over a third of PLHIV with RTRI recent results in Eswatini. These findings can be used to improve the accuracy of models used to estimate national epidemic trends. Possible mitigation strategies could include strengthening client‐centred care, better pre‐test counselling, and integrated data systems to confirm previous HIV care status and minimize unnecessary HIV testing of PLHIV who are already on ART.

**Abbreviations**
AIDS: acquired immunodeficiency syndrome
ANC: antenatal clinic
ART: antiretroviral therapy
ARV: antiretroviral
aOR: adjusted odds ratio
COVID‐19: Coronavirus disease 2019
CWC: child welfare clinic
ED: emergency department
EHRIS: Eswatini HIV‐1 Recent Infection Surveillance
FP: family planning
HIV: human immunodeficiency virus
HTS: HIV testing services
IP: inpatient
OPD: outpatient department
OR: odds ratio
PEPFAR: U.S. President's Emergency Plan for AIDS Relief
PLHIV: people living with HIV
PNC: postnatal clinic
RITA: recent infection testing algorithm
RTRI: rapid test for recent infection
STI: sexually transmitted infection
VCT: voluntary counselling and testing
VL: viral load
VLS: viral load suppression
VMMC: voluntary medical male circumcision
WHO: World Health Organization

## 1 Background

Eswatini is rapidly approaching HIV epidemic control. Of people living with HIV (PLHIV) aged 15 years and older, 93.7% know their HIV status, and of those, 97.3% are on antiretroviral therapy (ART) and 96.2% with viral load (VL) suppression [1, 2]. Measuring progress to global and national 95‐95‐95 targets often relies on infrequent, resource‐intensive population‐level studies and modelling. Reliable estimation of new HIV diagnoses identified through routine HIV testing services (HTS) is one of the essential measures for models, such as the UNAIDS Spectrum model for generalized epidemics.

Prior HIV diagnosis and ART use are not well captured in routine programme data and introduce uncertainty to estimates of new diagnoses [3, 4, 5]. Double counting new diagnoses in programmatic data due to retesting is likely common and could contribute to overestimation of new HIV diagnoses. Non‐disclosure of prior HIV diagnosis and ART use in routine HTS may also result in unnecessary HIV testing for PLHIV, potentially providing inaccurate results or inappropriate care for their needs, and is not recommended by the World Health Organization (WHO) [6]. Research has shown that some individuals conceal prior HIV diagnosis and ART use during pre‐test screening in clinical and research settings [7, 8, 9, 10]. Evidence suggests that non‐disclosure of HIV status or prior ART use is higher among younger age groups and men, although pregnant women are often excluded [7, 8, 9, 11].

Studies reporting on retesting in routine HTS often focus on pre‐treatment retesting, have limited scope or generalizability, and so estimates of retesting lack consensus [12]. Existing literature rarely distinguishes between individuals retesting before ART initiation and those already on ART who retest without disclosing their treatment status. Quantifying retesting among PLHIV on ART provides important insight into programme inefficiencies and distorted estimates of new HIV diagnoses, which are vital for improving epidemic control strategies. Estimates of undisclosed ART use in routine testing settings remain limited, largely due to resource and logistical limitations that hinder biomarker testing for antiretrovirals (ARVs) among all newly diagnosed individuals.

In July 2019, the Eswatini HIV‐1 Recent Infection Surveillance (EHRIS) programme was established. This programme introduced a rapid test for recent infection (RTRI) that utilizes differences in antibody avidity to distinguish recent (<12 months) versus long‐term (≥12 months) acquisition. VL measurement was conducted to improve the accuracy of the RTRI classification. Those with VL <1000 copies/mL are assumed to be on ART and, therefore, classified as retesting. With these data, we can estimate the prevalence (or frequency) of retesting among those already on ART in routine HTS. This group does not include those who are retesting before initiating ART or after disengaging from ART, both of whom will likely have elevated VL.

## 2 Methods

### 2.1 Study Design

We conducted a retrospective cross‐sectional analysis of data collected through EHRIS to examine the prevalence of retesting among those with RTRI recent results. We examined factors correlated with retesting, including demographic and behavioural characteristics, HIV service characteristics and level of COVID‐19 stay‐at‐home restrictions when HIV testing occurred.

### 2.2 Setting

Between July 2019 and February 2024, HIV‐1 Recent Infection Surveillance testing was offered in 384 facility‐ and community‐based locations in all four regions of Eswatini as part of routine HTS. Of these, 208 testing locations reported at least one RTRI recent result and were included in this analysis. Participants from the following settings which implemented a recent infection testing algorithm (RITA) were included: voluntary counselling and testing (VCT), antenatal clinic (ANC), sexually transmitted infection clinic (STI), community (both mobile outreach and community index testing), facility‐based index testing, child welfare clinic (CWC), emergency department (ED), family planning clinic (FP), maternity, outpatient department (OPD), postnatal clinic (PNC) and others.

### 2.3 Study Procedures

Adults aged 15 years and older with an HIV‐positive screening test as part of the national HIV testing algorithm in routine HTS were offered RTRI by opt‐out participation. RTRI was conducted in parallel with the second confirmatory HIV test. RITA in Eswatini included RTRI, using the Asante HIV‐1 Rapid Recency Assay (Sedia BioSciences, Portland, OR), followed by VL testing (Roche COBAS AmpliPrep/COBAS TaqMan HIV‐1) on all RTRI recent samples. Those with RTRI long‐term results did not undergo VL testing. All participants completed a basic, unvalidated demographic‐behavioural questionnaire that captured HIV testing history, sex, age, current pregnancy, marital status, education level, place of residence, testing date and location, and HIV risk behaviour (self‐reported identification with a key‐population, number of sexual partners and history of sexual violence). (Supporting Information).

We analysed the characteristics associated with retesting among individuals with a presumed new HIV diagnosis and VL result available from the RITA. Individuals who tested RTRI recent with VL ≥1000 copies/mL were classified as likely newly diagnosed with HIV, and those with VL <1000 copies/mL were considered as already being on ART and, therefore, retesting. We did not analyse retesting among individuals with RTRI long‐term results, as VL was not collected for this population.

EHRIS was a routine public health surveillance activity implemented by the Eswatini Ministry of Health. The programme was reviewed by the CDC, Eswatini Health and Human Research Review Board and Columbia University Institutional Review Board, and deemed to be non‐research public health surveillance. Participation was voluntary and offered on an opt‐out basis, with verbal informed consent and a waiver of written consent.

### 2.4 Statistical Methods

We used Rao−Scott Chi^2^ tests to compare the characteristics of individuals included in the retesting analysis against those who were excluded from the analysis (individuals with or without VL results, respectively). Analysis used HIV testing locations (health facility or major area of community testing) as the clustering unit. Bivariable logistic regression models assessed for unadjusted odds of retesting within population sub‐groups, with robust standard errors to account for clustering within HIV testing locations. Multivariable regression modelling with stepwise selection was used for the final adjusted model. We examined sex and pregnancy; age group (above and below 30 years); region of current residence (Hhohho, Manzini, Shiselweni and Lubombo); marital status (never married vs. ever married or living together), education level attained (none or primary and secondary, high school, tertiary education), timing of HIV testing relative to COVID‐19 stay‐at‐home restrictions (level 0 = no restriction, 1 July 2019–26 March, 2020 and 10 December 2021–28 February 2024; level 1 = stay‐at‐home recommended, 18 August 2020–4 January 2021 and 27 April 2021–5 June 2021; level 2 = stay‐at‐home required, 27 March 2020–17 August 2020, 5 January 2021–26 April 2021 and 6 June 2021–10 December 2021) [13], testing site, HIV testing history, prior use of HIV self‐test kits, self‐reported population risk status, number of sexual partners and history of sexual violence. We grouped HTS settings into five groups based on similar populations served: (1) OPD, emergency, VCT; (2) facility‐based index testing; (3) community‐based testing; (4) ANC, CWC, PNC, maternity, FP, STI; and (5) other/miscellaneous. During sensitivity analyses, we stratified the fully adjusted model by sex to examine the differences in factors contributing to retesting by sex. All analyses were conducted using SAS (version 9.4; SAS Institute Inc., Cary, NC, USA).

## 3 Results

### 3.1 Participants

**FIGURE 1:**
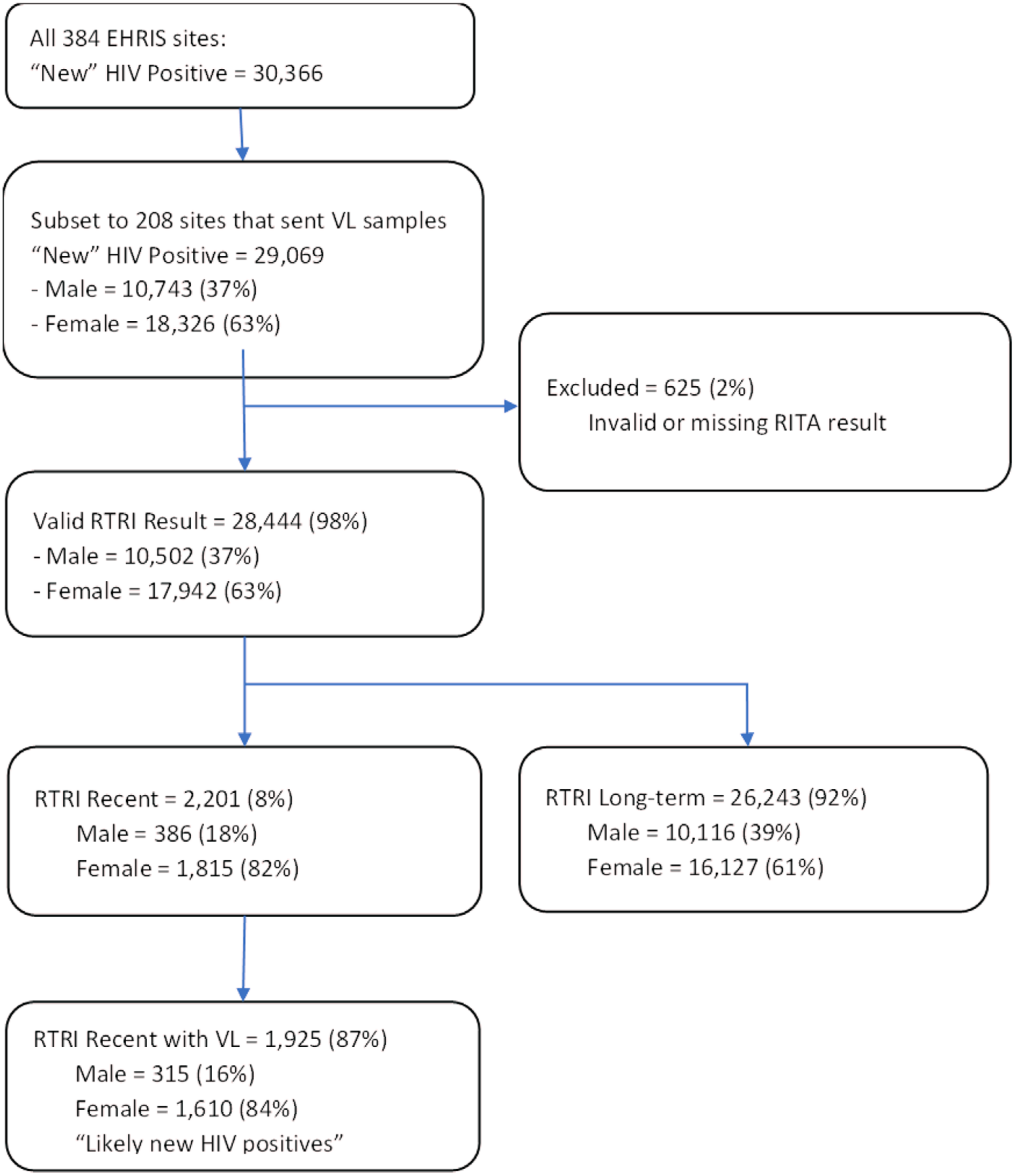
Eswatini HIV‐1 Recent Infection Surveillance participation cascade, July 2019–February 2024.

Between 1 July 2019, and 28 February 2024, 29,069 individuals tested HIV‐positive and were offered RITA as part of EHRIS in 208 testing locations nationwide. This represents 95.7% of 30,366 who tested HIV‐positive across all 384 testing locations. Of those, 28,444 (97.8%) had an RTRI result, and 625 (2.1%) were excluded due to invalid or missing results. Among those with valid RTRI results, 2201 (7.7%) tested RTRI recent and were eligible for VL, and 26,243 (92.3%) tested as long‐term. In total, 1925 (87.5%) underwent VL testing (Figure 1). Of those, 781 (40.6%: 95% CI, 37.1–44.1) had VL <1000 copies/mL and were classified as retesting (Table 2).

### 3.2 Characteristics of Individuals Included in the Retesting Analysis (PLHIV With RTRI Recent With VL Results) Versus Excluded (PLHIV With RTRI Long‐Term Result)

Compared to those with RTRI long‐term results, the 1925 individuals with RTRI recent and VL results were more likely to be both non‐pregnant females (60.2% vs. 47.6%, *p*<0.0001) and pregnant females (23.9% vs. 13.9%, *p*<0.0001); younger (aged 15–29 years, 73.8% vs. 43.7%, *p*<0.0001) and never married (76.6% vs. 65.2%, *p*<0.0001). RTRI recent with VL results, compared to RTRI long‐terms, were also more likely to have been tested for HIV in ANC, CWC, PNC, maternity, FP, STI (17.5% vs. 8.8%, *p*<0.0001). Further, RTRI recent with VL results, compared to RTRI long‐terms, were more likely to have been diagnosed prior to March 2020 (26.9% vs. 16.3%, *p*<0.0001), were more likely to have reported having received an HIV test in the past (71.6% vs. 55.8%, *p*<0.0001) or used an HIV self‐test kit (16% vs. 9.6%, *p*<0.0001). RTRI recent with VL results were less likely to have reported having no sexual partners in the past 12 months (1.7% vs. 5.2%, *p*<0.0001) and were more likely to have experienced sexual violence (2.7% vs. 1.3%, *p* = 0.0001). There were no observable differences in educational attainment, region of residence or identification as a population at risk (Table 1).

**TABLE 1:** Characteristics of PLHIV in Eswatini HIV‐1 Recent Infection Surveillance data with RTRI recent and VL results and RTRI long‐term without VL results who completed the Recent Infection Surveillance testing between July 2019 and February 2024.

	PLHIV with RTRI recent and VL result			PLHIV with RTRI long‐term without VL results	
	*n*	% (95% CI)	*n*	% (95% CI)	*p*‐value
Total	1925		26,134		
**Sex**					<0.0001
Female, non‐pregnant	1157	60.2 (56.3−64)	12,490	47.6 (44.7−50.6)	
Female, pregnant	453	23.6 (18.6−28.6)	3637	13.9 (10.4−17.4)	
Male	315	16.4 (14.4−18.5)	10,116	38.6 (36.5−40.7)	
**Age**					<0.0001
15−29 years	1419	73.8 (71.5−76)	11,443	43.7 (41.1−46.2)	
≥ 30 years	506	26.3 (24.1−28.6)	14,800	56.4 (53.9−59)	
**Marital status**					<0.0001
Never married	1465	76.6 (74.2−79.1)	17,007	65.2 (63.8−66.6)	
Married, living together, widowed, divorced	448	23.5 (21−25.9)	9079	34.9 (33.5−36.3)	
Missing	171				
**Education level attained**					0.24
None, primary	324	17.0 (15.0–19.0)	4721	18.1 (16.4−19.9)	
Secondary and above	1589	83.1 (81.1−85.1)	21,365	82 (80.2−83.7)	
Missing	171				
**Region of residence**					0.53
Hhohho	563	29.3 (19.4−39.2)	6987	26.7 (16.9−36.5)	
Lubombo	348	18.1 (12.2−24)	4441	17 (11−22.9)	
Manzini	684	35.6 (26.3−44.9)	10,124	38.6 (29.2−48.1)	
Shiselweni	290	15.1 (10−20.3)	3983	15.2 (9.1−21.3)	
Other (outside Eswatini)	40	2.1 (1.5−2.8)	708	2.7 (2.3−3.2)	
**HTS setting**					<0.0001
Index (facility‐based)	269	14.0 (9.4−18.7)	4448	17 (12.6−21.4)	
OPD, emergency, VCT	999	51.9 (43.6−60.3)	15,192	57.9 (49.1−66.8)	
Community	270	14.1 (7.6−20.5)	3113	11.9 (4.5−19.3)	
ANC, CWC, PNC, maternity, FP, STI	335	17.5 (12.6−22.3)	2302	8.8 (5.9−11.7)	
Other/miscellaneous	52	2.8 (1.3−4.2)	1188	4.6 (2.6−6.6)	
**Stay‐at‐home restriction level (start date)**					<0.0001
0 (1 July 2019–26 March 2020)	516	26.9 (22.2−31.6)	4274	16.3 (13.1−19.6)	
2 (27 March–17 August 2020)	168	8.8 (7.1−10.5)	2294	8.8 (7.3−10.3)	
1 (18 August 2020–4 January 2021)	191	10.0 (8.5−11.4)	2512	9.6 (8.7−10.5)	
2 (5 January–26 April 2021)	141	7.4 (6.1−8.7)	1967	7.5 (6.9−8.2)	
1 (27 April–5 June 2021)	36	1.9 (1.3−2.5)	715	2.8 (2.4−3.1)	
2 (6 June–10 December 2021)	200	10.4 (9−11.9)	2923	11.2 (10.3−12.1)	
0 (10 December 2021–28 February 2024)	673	35.0 (30.1−39.9)	11,558	44.1 (39.4−48.8)	
**Number of sexual partners in the past 12 months**					<0.0001
0	24	1.7 (1−2.4)	932	5.2 (3.9−6.6)	
1	909	61.6 (57.5−65.7)	11,000	61.3 (58.3−64.4)	
2+	543	36.8 (32.5−41.2)	6015	33.6 (29.7−37.5)	
Missing	9105				
**Ever tested for HIV**					<0.0001
No/don't know	547	28.5 (23.4−33.6)	11,525	44.3 (37.3−51.2)	
Yes	1374	71.6 (66.5−76.7)	14,545	55.8 (48.9−62.8)	
Missing	190				
**Ever tested with HIV self‐test**					<0.0001
No/don't know	1612	84.1 (81.7−86.4)	23,659	90.5 (89.1−91.9)	
Yes	307	16.0 (13.7−18.4)	2497	9.6 (8.2−11)	
Missing	98				
**Identify as key population**					0.02
No/don't know	1845	95.9 (94.2−97.6)	25,442	97 (96−98.1)	
Yes	80	4.2 (2.5−5.9)	800	3.1 (2−4.1)	
Missing	1				
**Experienced sexual violence**					0.0001
No/don't know	1874	97.4 (96.7−98.1)	25,905	98.8 (98.5−99)	
Yes	51	2.7 (2−3.4)	338	1.3 (1.1−1.6)	

Abbreviations: ANC, antenatal clinic; CWC, child welfare clinic; FP, family planning; IP, inpatient; L&D, labour and delivery; OPD, outpatient department; PLHIV, people living with HIV; PNC, postnatal care; RTRI, rapid test for recent infection; STI, sexually transmitted infection; VCT, voluntary counselling and testing; VL, viral load; VMMC, voluntary medical male circumcision.

### 3.3 Characteristics of Retesting on ART Versus Likely New HIV‐Positive Diagnoses

Overall, among 1925 individuals with RTRI recent results and available VL results, 781 (40.6%) had VL <1000 copies/mL, indicating they were likely already on ART and retesting, according to our definition (Table 2). Retesting was predominantly among non‐pregnant females (62.0%) followed by pregnant females (24.2%). Young adults aged 15–29 years represented 71.8% of the retesting population. Most had attained at least a secondary education (82.8%) and were never married (73.7%) (Table 2).

**TABLE 2:** Distribution of population sub‐groups among (a) people with a likely new HIV diagnosis, (b) people who were retesting and (c) the proportion of retesting within population sub‐groups among all with a likely new HIV diagnosis in Eswatini HIV‐1 Recent Infection Surveillance data with a VL result.

	Total	Likely new HIV‐positive diagnoses		Retesting			
	*n*	*n*	Distribution (a) % (95% CI)	*n*	Distribution (b) % (95% CI)	Proportion (c) % (95% CI)	*p*‐value
**Total**		1144	100	781	100	40.6 (37.1−44.1)	<0.0001
**Sex**							0.072
Female	1157	673	58.9 (54.5−63.3)	484	62.0 (57.5−66.5)	41.9 (37.7−46.1)	
Female, pregnant	454	263	23.0 (17.1−29)	190	24.4 (19.4−29.4)	42.0 (36.0–48.0)	
Male	315	208	18.2 (15.3−21.2)	107	13.8 (11.2−16.3)	34.0 (28.2−39.9)	
**Age**							0.158
15−29 years	1419	859	75.1 (72.1−78.2)	560	71.8 (68.2−75.3)	39.5 (35.4−43.6)	
≥ 30 years	506	285	25.0 (21.9−28)	221	28.3 (24.8−31.9)	43.7 (38.8−48.6)	
**Marital status**							0.01
Never married	1465	893	78.7 (75.6−81.7)	572	73.7 (70.6−76.8)	39.1 (35.2−43)	
Married, living together, widowed, divorced	448	243	21.4 (18.4−24.5)	205	26.4 (23.3−29.5)	45.8 (41.1−50.6)	
Missing	12						
**Education level attained**							0.7781
None, primary	324	190	16.8 (14.1−19.4)	134	17.3 (14.4−20.2)	41.4 (36.1−46.8)	
Secondary and above	1589	946	83.3 (80.7−86)	643	82.8 (79.9−85.7)	40.5 (36.5−44.5)	
Missing	12						
**Region of residence**							0.5314
Hhohho	563	345	30.2 (19−41.4)	218	28 (19.1−36.8)	38.8 (32−45.6)	
Lubombo	346	197	17.3 (11.1−23.5)	151	19.4 (12.9−25.8)	43.4 (36.5−50.4)	
Manzini	684	396	34.7 (24.6−44.7)	288	36.9 (28−45.8)	42.2 (37.7−46.6)	
Shiselweni	290	183	16.0 (10.3−21.8)	107	13.8 (8.8−18.7)	36.9 (31.3−42.6)	
Other (outside Eswatini)	40	23	2.1 (1.3−2.8)	17	2.2 (1.3−3.1)	42.5 (30.3−54.8)	
**Testing point**							0.0152
Index (facility)	269	174	15.3 (10.1−20.4)	95	12.2 (7.6−16.8)	35.4 (29.7−41.1)	
OPD, emergency, VCT	999	593	51.9 (43.1−60.7)	406	52.0 (43.1−61)	40.7 (36.2−45.2)	
Community	270	135	11.9 (5.7−18)	135	17.3 (9.8−24.8)	50.0 (43.1−57)	
ANC, CWC, PNC, maternity, FP, STI	335	203	17.8 (12−23.6)	132	17.0 (12.2−21.7)	39.5 (31.8−47.1)	
Other/miscellaneous	52	39	3.5 (1.5−5.4)	13	1.7 (0.8−2.6)	25 (15.2−34.9)	
**Stay‐at‐home restriction level (start date)**							<0.0001
0 (1 July 2019–26 March 2020)	516	339	29.7 (24.6−34.8)	177	22.7 (17.8−27.6)	34.4 (29.6−39.1)	
2 (27 March–17 August 2020)	168	93	8.2 (6.3−10)	75	9.7 (7.3−12)	44.7 (37.0−52.3)	
1 (18 August 2020–4 January 2021)	191	122	10.7 (8.9−12.5)	69	8.9 (6.7−11.1)	36.2 (29.0−43.4)	
2 (5 January–26 April 2021)	141	64	5.6 (4.2−7.1)	77	9.9 (7.6−12.3)	54.7 (45.0−64.3)	
1 (27 April–5 June 2021)	36	19	1.7 (1.0−2.4)	17	2.2 (1.2−3.2)	47.3 (32.4−62.1)	
2 (6 June–10 December 2021)	200	101	8.9 (7.2−10.6)	99	12.7 (10.2−15.3)	49.5 (41.7−57.4)	
0 (10 December 2021–28 February 2024)	673	406	35.5 (30.2−40.8)	267	34.2 (28.5−40)	39.7 (35.1−44.3)	
**Number of sexual partners**							0.6357
0	24	12	1.4 (0.7−2.1)	12	2.1 (0.8−3.3)	50 (31.6−68.5)	
1	909	552	62.1 (56.8−67.5)	357	60.9 (55.9−65.9)	39.3 (34.2−44.5)	
2+	543	325	36.6 (31.2−42.1)	218	37.2 (31.9−42.5)	40.2 (35−45.4)	
Missing	449						
**Ever tested for HIV**							0.017
No/don't know	547	300	26.3 (20.8−31.8)	247	31.8 (26.3−37.2)	45.2 (41−49.4)	
Yes	1374	842	73.8 (68.3−79.3)	532	68.3 (62.9−73.8)	38.8 (34.7−42.9)	
Missing	4						
**Ever tested with HIV self‐test**							0.3971
No/don't know	1612	965	84.7 (82−87.4)	647	83.1 (79.6−86.6)	40.2 (36.9−43.5)	
Yes	307	175	15.4 (12.7−18.1)	132	17 (13.5−20.5)	43 (35.5−50.6)	
Missing	6						
**Key population**							0.5309
No/don't know	1845	1100	96.2 (94.4−98)	745	95.4 (93−97.8)	40.4 (36.9−44)	
Yes	80	44	3.9 (2.1−5.7)	36	4.7 (2.3−7.1)	45 (30.7−59.4)	
**Experienced sexual violence**							0.0668
No/don't know	1874	1108	96.9 (95.9−97.9)	766	98.1 (97.2−99)	40.9 (37.4−44.4)	
Yes	51	36	3.2 (2.2−4.2)	15	2.0 (1.1−2.9)	29.5 (17.2−41.7)	

*Note*: PLHIV with likely new HIV‐positive diagnosis: RTRI recent with VL ≥1000 copies/mL. Retesting: RTRI recent with VL <1000 copies/mL.

Abbreviations: ANC, antenatal clinic; CWC, child welfare clinic; FP, family planning; IP, inpatient; L&D, labour and delivery; OPD, outpatient department; PNC, postnatal care; RTRI, rapid test for recent infection; STI, sexually transmitted infection; VCT, voluntary counselling and testing; VL, viral load; VMMC, voluntary medical male circumcision.

Manzini region had the highest proportion of retesting (36.9%), and the majority of retesting occurred in OPD, emergency or VCT (52.0%), followed by community HTS (17.3%). Retesting increased from 34.4% pre‐stay‐at‐home to 44.7%, 54.7% and 49.5% during three level 2 restriction periods before returning to 39.7% when all stay‐at‐home restrictions were removed. Most of those retesting reported having one sexual partner in the past 12 months (60.9%), self‐reported prior HIV testing (68.3%), but had not tested with an HIV self‐test (84.7%). Those who retested did not identify as a member of a population risk group and had not experienced sexual violence (Table 2).

### 3.4 Logistic Regression Analysis of Retesting

In bivariable analysis, factors associated with higher odds of retesting included female sex, both pregnant (OR [vs. male] 1.5, *p* = 0.03) and not (OR [vs. male] 1.4, *p* = 0.01), being married/living together, widowed or divorced (OR [vs. never married] 1.2, *p* = 0.01), tested in the community (OR [vs. facility index testing] 1.9, *p* = 0.0006) or during any of the level 2 stay‐at‐home restrictions between 27 March 2020 and 9 December 2021 (first period: OR [vs. before restrictions] 1.6, *p* = 0.02; second period: OR [vs. before restrictions] 2.4, *p*<0.0001; and third period: OR [vs. before restrictions] 1.9, *p* = 0.0002) and self‐reported prior HIV test history (OR [vs. never HIV tested] 1.4, *p* = 0.01) (Table 3). Age, education level attained, region, number of sexual partners in the past 12 months, identifying as a population at risk and experience of sexual violence were not significantly associated with retesting.

**TABLE 3:** Unadjusted and adjusted odds ratio (OR) for retesting (RTRI recent and VL <1000 copies/mL) among participants of the Eswatini HIV‐1 Recent Infection Surveillance, July 2019–February 2024.

	Total (*N*)	PLHIV retesting (*n*)	Unadjusted OR (95% CI)	*p*‐value	Adjusted OR (95% CI)	*p*‐value
**Sex**						
Male	1157	484	Ref			
Female	453	190	1.4 (1.1−1.9)	0.02	1.6 (1.2−2.1)	0.002
Female, pregnant	315	107	1.5 (1.1−1.9)	0.03	1.8 (1.3−2.6)	0.001
**Age**						
15−29 years	1419	560	Ref			
≥ 30 years	506	221	1.2 (1−1.5)	0.098	1.3 (1.0−1.6)	0.13
**Marital status**						
Never married	1465	572	Ref			
Married, living together, widowed, divorced	448	205	1.4 (1.1−1.7)	0.01	1.8 (1.1−2.9)	0.03
Missing	12					
**Education level attained**						
None, primary	324	134	Ref			
Secondary and above	1589	643	1.0 (0.8−1.3)	0.77	1.0 (0.8−1.3)	0.77
Missing	12					
**Region of residence**						
Hhohho	563	218	Ref			
Lubombo	348	151	1.3 (1−1.6)	0.16	1.2 (0.9−1.5)	0.43
Manzini	684	288	1.2 (1−1.5)	0.23	1.1 (0.9−1.4)	0.50
Shiselweni	290	107	1.0 (0.7−1.3)	0.60	0.9 (0.7−1.2)	0.37
Other	40	17	1.2 (0.7−2.3)	0.64	1.3 (0.6−2.8)	0.62
**Testing point**						
Index (facility)	269	95	Ref			
OPD, emergency, VCT	999	406	1.3 (1−1.7)	0.11	1.2 (0.9−1.6)	0.39
Community	270	135	1.9 (1.3−2.6)	0.001	1.9 (1.4−2.8)	0.0007
ANC, CWC, PNC, maternity, FP, STI	335	132	1.2 (0.9−1.7)	0.30	1.1 (0.8−1.5)	0.97
Other/miscellaneous	52	13	0.7 (0.4−1.2)	0.15	0.6 (0.3−1.2)	0.14
**Stay‐at‐home restriction level (start date)**						
0 (1 July 2019–26 March 2020)	516	177	Ref			
2 (27 March–17 August 2020)	168	75	1.6 (1.1−2.3)	0.02	1.7 (1.2−2.5)	0.005
1 (18 August 2020–4 January 2021)	191	69	1.1 (0.8−1.6)	0.65	1.2 (0.9−1.7)	0.40
2 (5 January–26 April 2021)	141	77	2.4 (1.6−3.4)	<0.0001	2.6 (1.7−3.8)	<0.0001
1 (27 April–5 June 2021)	36	17	1.8 (0.9−3.4)	0.12	1.9 (1−3.8)	0.08
2 (6 June–10 December 2021)	200	99	1.9 (1.4−2.7)	0.0002	2.1 (1.5−3)	<0.0001
0 (10 December 2021–28 February 2024)	673	267	1.3 (1.0−1.6)	0.06	1.4 (1.1−1.8)	0.04
**Number of sexual partners**						
0	24	12	Ref			
1	909	357	0.7 (0.3−1.5)	0.29	0.8 (0.4−1.9)	0.57
2 or more	543	218	0.7 (0.3−1.6)	0.34	1.0 (0.7−1.7)	0.97
Missing	449					
**Ever tested for HIV**						
No/don't know	547	247	Ref			
Yes	1374	532	1.4 (1.1−1.6)	0.01	1.3 (1.1−1.6)	0.03
Missing	4					
**Ever tested with HIV self‐test**						
No/don't know	1612	647	Ref			
Yes	307	132	1.2 (0.9−1.5)	0.35	1.1 (0.9−1.5)	0.50
Missing	6					
**Population at risk**						
No/don't know	1845	745	Ref			
Yes	80	36	1.3 (0.8−1.9)	0.41	1.0 (0.6−1.7)	0.98
**Experienced sexual violence**						
No/don't know	1874	766	Ref			
Yes	51	15	0.7 (0.4−1.2)	0.10	0.6 (0.4−1.2)	0.09

Abbreviations: ANC, antenatal clinic; CWC, child welfare clinic; FP, family planning; IP, inpatient; L&D, labour and delivery; OPD, outpatient department; OR, odds ratio; PNC, postnatal care; STI, sexually transmitted infection; VCT, voluntary counselling and testing; VMMC, voluntary medical male circumcision.

On multivariable analysis, pregnant women had 80% higher odds, and non‐pregnant women 60% higher odds of retesting compared to males (aOR 1.8, *p* = 0.001 and aOR 1.6, *p* = 0.002, respectively). Individuals who reported being married/living together/widowed/divorced had 80% higher odds of retesting than those who had never married (aOR 1.8, *p* = 0.03) (Table 3). Retesting was 90% more likely if individuals tested in community‐based versus facility‐based settings (aOR 1.9, *p* = 0.0007), and 30% more likely if they self‐reported prior HIV testing compared with those who had not (aOR 1.3, *p* = 0.03). Individuals who were HIV‐tested during level‐2 stay‐at‐home restrictions had 1.7‐fold, 2.6‐fold and 2.1‐fold higher odds of retesting compared to individuals tested during no restrictions (aOR 1.7, *p* = 0.005; 2.6, *p*<0.0001; and 2.1, *p*<0.0001, respectively).

## 4 Discussion

Retesting was detected in over one‐third of PLHIV with RTRI recent results captured in HIV‐1 Recent Infection Surveillance data in Eswatini between July 2019 and February 2024. The 40.6% frequency of retesting in our study population is roughly consistent with estimates from Eswatini, sub‐Saharan Africa and beyond [7, 8, 9, 10, 11, 12, 14, 15, 16]. However, direct comparison is hampered by non‐standardized “retesting” definitions, study populations or approaches. We measured retesting by virological suppression. Individuals retesting in our population can be assumed to be already on ART, a group not recommended by WHO for repeat testing [6]. As ART coverage among PLHIV increases globally over time, so will the proportion of previously diagnosed PLHIV who are undergoing retesting [3]. There is an increasing need to better understand this population.

Our findings align with population‐based studies where non‐disclosure of prior HIV diagnosis and ART use ranges from 11% to 39% [7, 8, 9, 11, 16]. These studies adopted various methods to detect retesting, including self‐report, VL and ARV detection using liquid chromatography‐mass spectrometry [7, 8, 9, 11, 16]. While these estimates are robust at the population level, such studies are seldom conducted more frequently than 5‐yearly, are costly to implement and are not representative of routine HTS populations included in our study. Our findings are similar to preliminary analysis of Recent Infection Surveillance data from Zambia, where baseline VL for all new HIV diagnoses found 37% viral load suppression (VLS) among PLHIV who were diagnosed with HIV in routine HTS [14]. In an analysis of retesting during HIV testing campaigns in Southern Mozambique, Fuente‐Soro et al. reported a retesting rate of 38.9% among presumed newly diagnosed PLHIV [12]. This study restricted the population to non‐pregnant adults aged ≥18 years who received HTS in one semi‐rural province and confirmed prior HIV diagnosis with an electronic patient tracking system [12]. Many other retesting studies either report retesting before ART initiation [15, 17, 18] or do not differentiate retesting ART history, and so are not comparable to our study population.

Clear patterns of subgroups that are likely to retest in routine HTS have yet to emerge in the literature. Zambia's Recent Infection Surveillance data reported a higher proportion of retesting among females (39.9%) and those aged ≥30 years (39.3%) [14]. Being pregnant is not a widely reported correlate of retesting; despite pregnant women being frequently targeted for repeat testing, they are often excluded from analyses [12, 15]. In population studies, retesting is not consistently associated with any age group [7, 8, 9]. Unlike many prior studies, our analysis includes community‐based testing sites and pregnant women.

Our study has some limitations. Our data includes a subset of PLHIV who retest through routine HTS, as only those testing RTRI recent were eligible for baseline VL testing, excluding those who retest but do not undergo RTRI testing or did not receive a VL test. In the same way retesting occurs in routine HTS, there is also a risk of double counting individuals who sought HIV testing at more than one facility during the study period, which impacts the entire study population. Without universal biomarker testing at baseline (VL or ARV detection), our findings lack generalizability to the HIV‐testing population. The excluded population likely includes PLHIV retesting for other important reasons, such as re‐engaging in care or treatment that are not captured by our testing algorithm. An exploratory study of adults initiating treatment in South Africa found that at least 45% had prior ARV exposure, including 21% identified only through records, often concealed due to negative provider reactions [19]. Second, our findings are representative of individuals who accessed routine HTS. Those who are historically missed by routine HTS, including younger adults and males, are likely underrepresented in our analysis [16]. Third, detecting retesting in our population relied on VLS and did not test for the presence of ARVs [8, 20, 21] nor review medical records [12]. So, the presence of elite controllers, though rare, may result in slight overestimation of retesting in our population [22, 23, 24]. Finally, observed differences may be multifactorial due to unequal offering of HIV testing across different populations, such as more testing among pregnant women.

Our study has several strengths. It is one of the few that reports on retesting among PLHIV who are sufficiently adherent to ART to be virally suppressed at the time of testing, which is a gap in available literature. This study makes a unique contribution by using baseline VL through Recent Infection Surveillance to estimate retesting among PLHIV on ART in routine settings. To our knowledge, this is the first study to use a reliable biomarker test, rather than self‐report, in routine testing to estimate retesting for PLHIV currently on ART, offering a more accurate measure of retesting than self‐report methods. Our study includes a larger and more inclusive sample than many previous similar studies. Our study included data from 208 high‐volume HIV testing settings, including both facility and community‐based sites countrywide, that collectively identified 95% of adults who tested positive for HIV in all four regions nationally. We also analysed data across the entire timeline of evolving “stay‐at‐home” restrictions adopted in Eswatini between March 2020 and December 2021. We found that periods of greater restriction corresponded with significantly higher odds of retesting and highlighted the importance of considering policy‐level disruptions on HIV testing patterns, and potential adjustments to be considered when analysing HIV testing data from these periods. Finally, our study included a more inclusive population than comparable studies, as our data included all newly diagnosed individuals aged 15 years and above and did not restrict participation based on sex, pregnancy or breastfeeding status.

Understanding the volume of retesting in routine HTS has several important public health implications. PLHIV on ART who seek retesting require different support than both newly diagnosed PLHIV or returning to care. Healthcare workers must be aware of and screen for prior ART use during pre‐test screening to ensure appropriate psychosocial support for PLHIV based on their stage of care, as well as avoid unnecessary use of limited resources or provider time. Retesting while on ART is not recommended by WHO. It is important to distinguish between those who have not initiated or have discontinued ART, as seen in Eswatini [19]. Retesting for these reasons represents important opportunities to engage or re‐engage PLHIV in care that should be discussed openly and supportively [6]. Knowing which PLHIV are more likely to engage in retesting can assist programmes in implementing targeted quality improvement efforts to better identify and serve PLHIV already on ART. Reasons for retesting were not examined in our study, and available evidence suggests that they can be driven by factors on both the client‐side and provider‐led, though these seldom differ by ART status [15, 17, 18, 25, 26]. Client motivations for retesting could include fear of negative healthcare worker reactions, disbelief of HIV diagnosis or doubting results, low perceived risk, or belief that ART may cure HIV. Pre‐test screening that omits prior ART status may lead HTS providers to incorrectly assume the client is ART‐naive and to offer testing accordingly [27]. More research is needed, however, if explicitly asked about prior ART use upfront, whether the client may be encouraged to share this information and not undergo unnecessary retesting.

When considering modelled estimates of HIV testing and awareness of HIV status, biomarker‐based estimates of retesting from routine HIV testing may be a useful parameter. The UNAIDS Spectrum model, for example, which in generalized epidemics such as Eswatini uses inputs from ANC sentinel surveillance and population‐based estimates to estimate new diagnoses and more recently includes case reporting in countries with strong case reporting and vital registration, may overestimate new diagnoses if people with a prior HIV diagnosis or on ART are not well captured [5]. The Shiny90 model, used to estimate HIV testing with UNAIDS Spectrum as the basis, attempts to correct the over‐counting of positive tests by using available empirical data to account for potential differences in testing rates as a function of HIV status and past testing history, including for PLHIV who have a prior HIV diagnosis and are not on treatment, and those who are on ART [4]. In both examples, the precision of the volume of retesting among PLHIV on ART in routine HTS can improve the accuracy of the modelled estimates of HIV testing progress. Retesting in routine surveillance or ANC data potentially leads to overestimating progress towards testing targets. As ART coverage rises, a higher proportion of new diagnoses will be already on ART. So, it will be increasingly important to have accurate estimates of the volume of retesting occurring to determine where the gaps remain. Building empirical evidence and strengthening surveillance systems to continuously monitor this retesting in this population will improve the accuracy of estimates of new HIV diagnoses.

## 5 Conclusions

Our findings demonstrate a need for more appropriate testing that might be addressed by providing person‐centred care in HTS and ART care that aims to ensure individuals are getting the right service at the right time according to their stage of treatment without having to undergo unnecessary HIV testing and minimize demand for retesting among clients already on ART. There is a role for improved pre‐test counselling and screening, as well as an important role for robust HIV surveillance systems, electronic medical records and case surveillance systems to help track PLHIV throughout various encounters. This will enable HIV services to streamline data systems, reduce duplication across testing and ART records, and strengthen care continuity for PLHIV (Supporting Information).

## Author Contributions

Claire Steiner conceived and wrote the first draft of the manuscript and conducted statistical analyses. Claire Steiner, Suzue Saito, Harriet Nuwagaba‐Biribonwoha, Eugenie Poirot, Samkelo Simelane, Dumile Sibandze, Lenhle Dube, Vusie Lokotfwako, Munyaradzi Pasipamire and Cebisile Ngcamphalala contributed to study investigation, methodology and validation of the study data. Jessica Justman and Harriet Nuwagaba‐Biribonwoha acquired funding for the study. Claire Steiner, Suzue Saito, Harriet Nuwagaba‐Biribonwoha, Eugenie Poirot, Samkelo Simelane, Munyaradzi Pasipamire and Cebisile Ngcamphalala administered the project and resources and supervised the study. All authors reviewed and edited the final version of the manuscript.

## Funding

This research was supported by the United States President's Emergency Plan for AIDS Relief (PEPFAR) through the US Centers for Disease Control and Prevention under the terms of cooperative agreements NU2GGH002171 and U2GGH002291.

## Conflicts of Interest

The authors declare that they have no conflicts of interest related to the content of this article. No financial, personal, or professional relationships with other people or organizations could inappropriately influence work. All authors have contributed significantly to the research, analysis, and writing of this manuscript and have reviewed and approved the final version. There are no competing interests that could have influenced the results or interpretations presented in this study. This research was conducted independently and was not influenced by external funding sources or sponsors.

## Disclaimer

The findings and conclusions in this article are those of the authors and do not necessarily represent the official positions of the funding agencies.

## Supporting information




**Supporting File**: jia270187‐sup‐0001‐Appendix.pdf.

## Data Availability

The data that support the findings of this study may be available from the Ministry of Health of the Kingdom of Eswatini upon reasonable request.
