# Supplementary material for: Have We Met Before? Findings on HIV Retesting in Routine Healthcare Settings, Using Eswatini HIV‐1 Recent Infection Surveillance Data
Source: J Int AIDS Soc. 2026 Sep 16;29(9):e70187. doi: 10.1002/jia2.70187 (PMC13579381; doi:10.1002/jia2.70187)
Supplement: Supplementary file 1 [file JIA2-29-e70187-s001.pdf]

Eswatini HIV Recent Infection Surveillance (EHRIS)
Data Collection Form-English

Part I: Complete for all clients age 15 years and older with a new HIV diagnosis.

SECTION A: HIV TESTING SERVICES INFORMATION

1. HIV Testing Facility/Site:

2. Point of HIV testing service where the case was diagnosed:

OPD		Inpatient		STI		VMMC		FP		TB	
ANC		Maternity/L&D		PNC		CWC		VCT		HBHTC	
		Mobile/Outreach		Emergency		Other		If Other, specify:			

Index

Yes		No	

3. If testing was performed through mobile or outreach testing, what is the location of testing?

a. Region		b. Inkhundla		c. Major Area		d. Sub-Area	

4. Age	5. Sex		6. Date of Test	7. HIV Test Result					
	Male	Female	dd/mm/yyyy	Determine		UniGold		Accepted Asante	
				Reactive		Reactive		Yes	
				Nonreactive		Nonreactive		No	

8. If Asante was not done, what was the reason?

Client opted out/refused		Client ineligible		No trained HTS provider available		Missing testing supplies		Other		If other, Specify:	
**9. Did client opt-out of record review?**		Yes (Client opted out, do not review records)		No (Client did NOT opt out, records can be reviewed)							

Part II: Complete for all clients that accept Asante.

SECTION B: CLIENT IDENTIFIERS

(Information from this section will be used to uniquely identify a person and link their health information over time. All personally identifiable information, including full name and national ID number, will be removed from the analysis database.)

1. Full Name			2. CMIS number	
First name	Middle Name	Surname		Not available

3. HTS number		4. National identification number		5. EHRIS number
	Not available		Not available	

SECTION C: CLIENT HIV TESTING INFORMATION

1. Asante test kit lot number:	2. Asante RTRI result:											
	Control (C) line seen				Verification (V) line seen				Long-term (LT) line seen			
3. Asante test kit expiry date:	Yes		No		Yes		No		Yes		No	
dd/mm/yyyy	If No V or C line seen, collect blood sample for repeat RTRI testing and VL testing in the laboratory								If No LT line seen, collect blood sample for VL testing in the laboratory			

4. Before today, had you ever been tested for HIV at a health facility or by a community-based testing program?

Yes		No		Don't know		Refused to respond	

a. If yes, what was the result?

HIV-negative		HIV-positive		HIV-inconclusive		Don't know		Refused to respond	

Columbia University IRB
IRB-AAAS3117 (Y02M00)
IRB Approval Date: 04/09/2020
For use until modified or study is closed

EHRIS data collection form (English)

**b. Date of test** dd/mm/yyyy							
**5. Have you tested yourself for HIV using a self-test kit in the last 12 months (e.g., oral self-test kit)?**							
Yes		No		Don't know		Refused to respond	

**a. If yes, what was the result?**		**b. Where did you receive the self-test kit from?**			
HIV-negative		Health facility		Community outreach	
HIV-positive		Index partner		Private pharmacy	
Don't know		Index family member		Other	
Refused to respond		Church		If other, specify:	

**6. Have you ever taken or are you currently taking ARVs (ART) for treating diagnosed HIV infection?**							
Yes		No		Don't know		Refused to respond	
**7. If the client is female, is she pregnant?** *Tick appropriately below*							
Yes		No		Don't know		Refused to respond	

SECTION D: CLIENT DEMOGRAPHIC INFORMATION

**1. Nationality**	Swazi		Non-Swazi		If non-Swazi, specify:

**2. Current Residence**		**3. Is your permanent residence the same your current residence?**	
a. Country		Yes / No	If no, specify permanent residence:
a. Region		a. Country	
b. Inkhundla		c. Region	
d. Major Area		e. Inkhundla	
f. Sub-Area		g. Major Area	
		h. Sub-Area	

**4. Marital status**		**5. Education (highest level completed)**	
Never married		None	
Married		Primary	
Living together		Secondary	
Widowed		High school	
Divorced/separated		Tertiary	

SECTION E: BEHAVIOR QUESTIONS

**1. How many sexual partners have you had in the past 12 months?**									
**2. How often have you used condoms when having sex in the past 12 months?**									
Always		Most of the time		Sometimes		Rarely		Never	
Not applicable		Refused to respond		Don't know					

**3. Have you injected drugs and shared needles, syringes, or other drug equipment with others in the past 12 months?**							
Yes		No		Don't know		Refused to respond	
**4. In the past 12 months, have you been forced to have sex against your will?**							
Yes		No		Don't know		Refused to respond	
**5. In the past 12 months, have you had sex in exchange for money as your primary source of income?**							
Yes		No		Don't know		Refused to respond	
**6. [If Male] In the past 12 months, have you had sex with a partner of the same sex?**							
Yes		No		Don't know		Refused to respond	

SECTION F: STAFF DETAILS

**1. Name of person completing form:**
**2. Designation of person completing form**

Columbia University IRB
IRB-AAAS3117 (Y02M00)
IRB Approval Date: 04/09/2020
For use until modified or study is closed

EHRIS data collection form (English)

HTS counselor		Nurse		Data clerk		Other		If other, specify:

Columbia University IRB
IRB-AAAS3117 (Y02M00)
IRB Approval Date: 04/09/2020
For use until modified or study is closed
